# Wearable ECG-PPG Deep Learning Model for Cardiac Index-Based Noninvasive Cardiac Output Estimation in Cardiac Surgery Patients

**DOI:** 10.3390/s26020735

**Published:** 2026-01-22

**Authors:** Minwoo Kim, Min Dong Sung, Jimyeoung Jung, Sung Pil Cho, Junghwan Park, Sarah Soh, Hyun Chel Joo, Kyung Soo Chung

**Affiliations:** 1 MEZOO Co., Ltd., 200, Gieopdosi-ro, Jijeong-myeon, Wonju-si 26354, Republic of Korea; mwkim@me-zoo.com (M.K.); jmjung@me-zoo.com (J.J.); spcho@me-zoo.com (S.P.C.); jhpark@me-zoo.com (J.P.); 2 Division of Pulmonology and Critical Care Medicine, Department of Internal Medicine, Institute for Innovation in Digital Healthcare, Yonsei University College of Medicine, Seoul 03722, Republic of Korea; mdsung@yuhs.ac; 3 Anaesthesia and Pain Research Institute, Department of Anaesthesiology and Pain Medicine, Yonsei University College of Medicine, Seoul 03722, Republic of Korea; yeonchoo@yuhs.ac; 4 Division of Cardiovascular Surgery, Department of Cardiovascular and Thoracic Surgery, Yonsei University College of Medicine, Seoul 03722, Republic of Korea; 5 Institute for Innovation in Digital Healthcare, Yonsei University College of Medicine, Seoul 03722, Republic of Korea

**Keywords:** wearable sensors, electrocardiography (ECG), photoplethysmography (PPG), cardiac output, cardiac index, deep learning, multimodal fusion, hemodynamic monitoring, cardiac surgery

## Abstract

**Highlights:**

**What are the main findings?**

- A lightweight wearable ECG–PPG fusion model estimated cardiac index (CI) and cardiac output (CO), with the best performance observed for CI prediction.
- CI-based normalization improved agreement with thermodilution reference measurements, and indirect CO reconstruction met the predefined benchmark (percentage error, PE < 30%).

**What are the implications of the main findings?**

- Wearable ECG–PPG monitoring combined with deep learning may enable catheter-free, continuous trending of CO/CI in controlled perioperative settings.
- CI-normalized modeling may improve generalizability and support future noninvasive hemodynamic monitoring tools, pending external and ambulatory validation.

**Abstract:**

Accurate cardiac output (CO) measurement is vital for hemodynamic management; however, it usually requires invasive monitoring, which limits its continuous and out-of-hospital use. Wearable sensors integrated with deep learning offer a noninvasive alternative. This study developed and validated a lightweight deep learning model using wearable electrocardiography (ECG) and photoplethysmography (PPG) signals to predict CO and examined whether cardiac index-based normalization (Cardiac Index (CI) = CO/body surface area) improves performance. Twenty-seven patients who underwent cardiac surgery and had pulmonary artery catheters were prospectively enrolled. Single-lead ECG (HiCardi+ chest patch) and finger PPG (WristOx2 3150) were recorded simultaneously and processed through an ECG–PPG fusion network with cross-modal interaction. Three models were trained as follows: (1) CI prediction, (2) direct CO prediction, and (3) indirect CO prediction. The total number of CO = predicted CI × body surface area. Reference values were derived from thermodilution. The CI model achieved the best performance, and the indirect CO model showed significant reductions in error/agreement metrics (MAE/RMSE/bias; *p* < 0.0001), while correlation-based metrics are reported descriptively without implying statistical significance. The Pearson correlation coefficient (PCC) and percentage error (PE) for the indirect CO estimates (PCC = 0.904; PE = 23.75%). The indirect CO estimates met the predefined PE < 30% agreement benchmark for method-comparison; this is not a universal clinical standard. These results demonstrate that wearable ECG–PPG fusion deep learning can achieve accurate, noninvasive CO estimation and that CI-based normalization enhances model agreement with pulmonary artery catheter measurements, supporting continuous catheter-free hemodynamic monitoring.

## 1. Introduction

Cardiac output (CO), which is defined as the volume of blood pumped by the heart per minute, is a fundamental hemodynamic parameter used to assess cardiovascular function, guide therapeutic decisions, and predict clinical outcomes. CO is a central determinant of oxygen delivery and organ perfusion, and CO-/stroke-volume-guided hemodynamic management is widely used in perioperative care to individualize fluid, inotrope, and vasopressor therapy. Such goal-directed strategies have been evaluated in numerous randomized trials and systematic reviews, with reports of improved outcomes in selected surgical populations [1,2,3]. Accurate CO assessment is particularly important in patients with heart failure, cardiogenic shock, and sepsis and in those undergoing major cardiac surgery, where subtle changes in hemodynamics can significantly affect morbidity and mortality [4,5,6,7,8].

The pulmonary artery catheter (Swan-Ganz type) remains the clinical reference standard for CO measurements and provides high-fidelity hemodynamic data [9,10]. However, it requires invasive insertion into the pulmonary artery, is associated with procedure-related complications such as arrhythmias, infection, thromboembolism, and rarely, pulmonary artery rupture, and requires skilled personnel and intensive care resources [11,12]. Minimally invasive CO monitoring techniques based on arterial pulse wave analysis (e.g., PiCCO, LiDCO, and FloTrac) estimate stroke volume and CO from arterial pressure waveforms but still rely on arterial cannulation and are therefore unsuitable for long-term or out-of-hospital monitoring [13,14,15,16,17]. Noninvasive echocardiographic methods can estimate CO without vascular access, yet they are intermittent, operator-dependent, and constrained to in-hospital use even when assisted by artificial intelligence [18].

Recent advances in wearable sensing technology have opened up new possibilities for noninvasive hemodynamic monitoring [19]. Electrocardiography (ECG) and photoplethysmography (PPG) are representative biosignals that can be continuously acquired using compact low-power devices [20,21,22,23]. ECG reflects the electrical activation and timing of cardiac contraction, whereas PPG captures peripheral blood volume changes and pulse wave propagation [24,25]. The temporal relationship and morphological characteristics of these two signals are closely related to cardiovascular dynamics, including stroke volume and vascular compliance, and thus, have the potential to inform CO estimation when appropriately integrated [26,27,28]. Nevertheless, most wearable studies to date have focused on simpler metrics, such as heart rate, heart rate variability, or activity, and research on predicting advanced hemodynamic parameters like CO remains limited [29,30,31,32,33]. Recent studies have explored noninvasive CO estimation using PPG, either as a stand-alone input or combined with complementary signals [32,34,35]. Deep-learning models trained on large waveform databases have demonstrated the feasibility of estimating CO from PPG and multimodal physiologic waveforms, and pulse-wave-transit-time (PWTT)-based methods (e.g., estimated continuous cardiac output, esCCO) have also been evaluated with Bland–Altman agreement metrics in perioperative and exercise settings [36,37]. These studies motivate wearable, artifact-robust approaches, but performance varies across cohorts and clinical conditions, underscoring the need for modality fusion and careful physiological normalization.

In parallel, deep learning has demonstrated a remarkable performance in analyzing complex physiological waveforms. Multimodal architectures can learn the interactions between heterogeneous signals, enabling more informative fusion than traditional feature engineering [38,39,40]. In particular, attention-based ECG–PPG fusion models can leverage features such as pulse transit time and waveform morphology to detect subtle hemodynamic changes that are not captured by a single modality [41,42,43]. However, the optimal way to represent CO in such models—direct regression of CO versus indirect estimation via normalized indices—has not been fully explored.

In this study, we hypothesized that estimating cardiac index (CI), a body-size-normalized hemodynamic quantity, would reduce inter-subject variability compared with direct CO regression. Since CO is approximately CI × body surface area (BSA), learning CI first may encourage the network to capture physiological state more directly while handling anthropometric scaling explicitly via BSA, thereby improving robustness and generalization across patients. We developed a noninvasive CO prediction framework based on the deep learning fusion of wearable ECG and PPG signals collected from cardiac surgery patients equipped with a PAC. We compared two approaches: (1) direct CO prediction and (2) indirect prediction via cardiac index (CI = CO/body surface area), where the CO was reconstructed by multiplying the predicted CI by the body surface area. We also designed the model with lightweight architecture suitable for real-time implementation on wearable or edge-computing platforms. We hypothesized that CI-based indirect prediction would provide better agreement with invasive CO measurements than direct CO prediction and thus offer a more robust solution for continuous catheter-free hemodynamic monitoring.

## 2. Materials and Methods

### 2.1. Study Design and Population

This prospective, single-center observational study was conducted at Severance Hospital, Yonsei University College of Medicine, Seoul, Republic of Korea. Adult patients (≥18 years) scheduled to undergo cardiac surgery requiring PAC for perioperative hemodynamic monitoring were eligible for inclusion.

Patients were excluded if they (1) declined to provide informed consent, (2) were unable to wear the wearable devices used in this study (e.g., due to skin conditions, anatomical limitations, or intolerance), (3) were expected to be unable to complete the data acquisition protocol after enrollment, or (4) had language barriers that could interfere with understanding the study information or communication with the research team.

For each participant who provided written informed consent before surgery, data acquisition was initiated after the induction of general anesthesia, initiation of mechanical ventilation, and placement of the PAC. The PAC was connected to the HemoSphere monitoring system (Edwards Lifesciences LLC, Irvine, CA, USA), and the correct transmission of hemodynamic data to the VitalDB platform (Seoul National University Hospital, Seoul, Republic of Korea) was confirmed before starting the recording session [44].

During the hemodynamic stabilization period following catheter insertion, the HiCardi+ single-lead ECG patch (MEZOO Co., Ltd., Wonju-si, Gangwon-do, Republic of Korea) was attached to the left parasternal area between the second and fourth intercostal spaces, and the WristOx2 Model 3150 finger PPG sensor (Nonin Medical, Inc., Plymouth, MN, USA) was placed on the third finger of the left hand to ensure stable signal acquisition. ECG and PPG signals were continuously recorded for at least 10 min and up to 20 min, respectively, per subject.

The study protocol was approved by the Institutional Review Board of Severance Hospital (IRB No. 4-2024-0227) and was conducted in accordance with the principles of the Declaration of Helsinki. All participants provided written informed consent prior to enrollment after receiving an explanation of the study’s purpose and procedures. ECG- and PPG-based wearable devices were provided by the respective manufacturers without any financial compensation.

### 2.2. Wearable Devices and Signal Acquisition

Two wearable devices were used for simultaneous ECG and PPG acquisition: a single-lead ECG chest-patch (HiCardi+, MEZOO Co., Ltd., Wonju-si, Gangwon-do, Republic of Korea) and a wrist-worn pulse oximeter with a finger sensor (WristOx2 Model 3150, Nonin Medical, Inc., Plymouth, MN, USA). These devices were selected to represent compact, low-power, wearable platforms already used in clinical and ambulatory monitoring. The detailed hardware specifications of both devices are provided in Supplementary Tables S1 and S2.

The HiCardi+ device is a wireless, smart patch–based ECG monitor that continuously records single-lead ECG from the chest. In routine clinical use, it also supports respiration, skin temperature, posture/activity, and step/movement monitoring and provides real-time ECG streaming with automatic detection of multiple arrhythmia types. In addition, HiCardi+ incorporates a high-performance R-peak detection algorithm that utilizes the geometric angle between consecutive ECG samples. This algorithm is robust against variations in the QRS complex morphology, amplitude fluctuations, and baseline drift, thereby ensuring stable and accurate ECG feature extraction [45]. In this study, only a single-lead ECG waveform was used for the model development. The ECG signals were sampled at 250 Hz with a 16-bit resolution and transmitted via Bluetooth Low Energy to a gateway device.

The WristOx2 Model 3150 is a wrist-worn pulse oximeter that measures PPG waveforms through an infrared/red finger sensor and concurrently derives the oxygen saturation (SpO_2_) and pulse rate. The device incorporates motion-tolerant signal processing (PureSAT^®^ (Nonin Medical, Inc., Plymouth, MN, USA)) and is clinically validated for continuous SpO_2_ monitoring in hospital and ambulatory environments. In this study, raw PPG waveforms were recorded at a sampling rate of 75 Hz with a 16-bit resolution and used as the primary input, whereas the derived SpO_2_ and pulse rate values were not used in the deep learning model.

During the recording session, both devices were synchronized at the start of the data acquisition. Data packets from the ECG patch and PPG oximeter were continuously transmitted via Bluetooth Low Energy to a smartphone, which served as a gateway, and the encrypted waveform data were uploaded to a secure research server. Device clocks and server timestamps were used to maintain temporal alignment between the ECG, PPG, and PAC measurements for subsequent preprocessing and model training.

### 2.3. Reference Cardiac Output and Cardiac Index Measurement

Reference CO values were obtained using a pulmonary artery catheter as the reference method for hemodynamic assessment. PAC-derived CO was measured using continuous thermodilution and recorded at 1 min timestamps via the HemoSphere monitoring system (Edwards Lifesciences, Irvine, CA, USA). These time-stamped CO values were exported through the VitalDB platform for synchronization with wearable ECG and PPG waveforms. CI was computed as CO normalized by body surface area (BSA).

Wearable ECG/PPG were segmented into 60 s windows with a 30 s shift. For each window, the reference time was defined as the segment midpoint (t_mid = t_start + 30 s). Each window was labeled using the PAC-derived CO (and corresponding CI) at the nearest 1 min timestamp (nearest-neighbor matching). Windows were excluded if the absolute timestamp difference exceeded ±30 s. Due to overlap, adjacent windows could share the same PAC reference value.

The following hemodynamic parameters were continuously collected via the PAC and HemoSphere system: average heart rate, CO, CI, stroke volume index, and mixed venous oxygen saturation. The distribution of the reference CO values across all study participants is presented in Supplementary Figure S1.

In this study, CI served as an intermediary parameter for the indirect prediction of CO. BSA was calculated using the Mosteller formula [46]:

(1) $$BSA\;{(m}^{2})=\sqrt{\frac{\mathrm{height}\;(\mathrm{cm})\times \mathrm{weight}\;(\mathrm{kg})}{3600}}$$

CI was defined as CO normalized by BSA:

(2) $$CI\;({L/min/m}^{2})=\frac{CO\;(L/min)}{BSA\;(m^{2})}$$

For the indirect CO prediction model, the deep learning network was trained to regress the CI from wearable ECG–PPG inputs, and the corresponding CO was reconstructed by multiplying the predicted CI by the patient-specific BSA:

(3) $${\hat{CO}}_{\mathrm{indirect}}=\hat{CI}\times BSA$$

This formulation allowed a direct comparison between the indirect CO estimates and the invasive reference CO values while leveraging the physiological normalization provided by the CI.

### 2.4. Signal Preprocessing and Segmentation

A total of 33 patients undergoing cardiac surgery were enrolled. Six patients were excluded because of excessive signal noise, incomplete waveform data, or device malfunction. After applying the signal quality and data completeness criteria, 27 patients were included in the final analysis, yielding 501 valid ECG–PPG segments (Supplementary Table S3).

Data were partitioned at the patient level to prevent information leakage across datasets. After applying signal quality and completeness criteria, the final dataset yielded 501 valid ECG–PPG segments. Patients were randomly assigned to training (16 patients, 302 segments, 59.26%), validation (5 patients, 108 segments, 18.52%), and testing (6 patients, 91 segments, 22.22%) sets. This approximately 6:2:2 split ensures sufficient data for model training while preserving independent patient cohorts for hyperparameter tuning and final performance evaluation. The training set was used for model fitting, the validation set for hyperparameter tuning and early stopping, and the test set for final performance evaluation.

For multimodal fusion, all ECG and PPG waveforms were resampled to a common sampling rate of 250 Hz to enable precise temporal alignment and facilitate learning of physiologically meaningful time-delay relationships (e.g., pulse transit time) in the cross-attention mechanism. Resampling was performed using Fourier-based interpolation (scipy.signal.resample), which operates in the frequency domain via FFT to preserve the original spectral content, phase relationships, and waveform morphology while preventing aliasing artifacts. Since PPG signals were bandpass-filtered to 0.5–4 Hz, all physiologically relevant information for CO prediction lies well below the original Nyquist frequency (37.5 Hz at 75 Hz sampling). Fourier resampling provides a higher temporal resolution for accurate inter-signal alignment without introducing morphological distortions that can occur with simpler interpolation methods.

The PPG signal was preprocessed using a 4th-order Butterworth band-pass filter with a passband of 0.5–4 Hz. This frequency range corresponds to a physiological heart rate range of approximately 30–240 beats per minute (0.5–4 Hz) and effectively attenuates low-frequency baseline drift and high-frequency noise while preserving the main pulse wave components. To further suppress residual high-frequency noise, a Savitzky–Golay filter (window size = 51 samples, polynomial order = 3) was applied. Amplitude normalization was not performed on PPG signals, as pulse amplitude contains physiologically meaningful information related to stroke volume and peripheral perfusion. All signals were acquired using the same calibrated device under standardized conditions, and signal quality was ensured through template-matching assessment rather than amplitude scaling. Unlike a simple moving average, the Savitzky–Golay filter performs local polynomial fitting, which smooths the waveform while preserving the position and amplitude of the peaks and the overall morphology of the PPG signal. In contrast, ECG signals contain diagnostically important fine structures, such as the P wave, QRS complex, and T wave; therefore, the original ECG waveform was used without additional filtering to avoid distortion of these features.

Signal quality was assessed using the NeuroKit2 library [47]. For the ECG, the QRS average method was adopted, which quantifies the quality of each heartbeat by comparing the morphology of the QRS complex around the detected R-peak with an average template. For the PPG, a template-matching method was used, in which a quality score was calculated by cross-correlating each detected pulse wave with a standard pulse template. The resulting quality scores were standardized as Z-scores, and segments with scores beyond ±2.0 standard deviations from the mean were considered to have significantly low signal quality and were excluded from further analysis. This threshold retains approximately 95% of the segments with typical quality while removing abnormal segments in a statistically principled manner.

After preprocessing and quality control, the signals were segmented into 60 s windows, corresponding to 15,000 samples at 250 Hz. The 60 s window length was selected because CO and CI are defined as per-minute physiological parameters (L/min and L/min/m^2^), making a 1 min measurement window the natural analysis unit. A 50% overlapping sliding-window strategy was applied (30 s stride), balancing data augmentation with segment independence. Greater overlap (e.g., 80%) caused excessive correlation and bias toward frequently represented CO/CI values, while less overlap would drastically reduce sample size in this limited patient cohort. For temporal alignment, ECG, PPG, and PAC data streams were synchronized using UNIX timestamps recorded at acquisition. For each 60 s wearable signal segment, PAC outputs whose timestamps fell within the segment’s time range were retrieved. Although the PAC monitoring system refreshes CO and CI internally on a 1 min computational cycle, the system delivers values as a 2 s interval streaming output. The reference CO and CI label for each segment was defined as the mean of all PAC outputs within the 60 s window, producing a temporally matched, robust ground-truth target for model training and evaluation.

Segments with missing reference values, null entries, or extremely small waveform amplitudes (indicating sensor detachment or measurement failure) were excluded from analysis. Only segments that met all quality and completeness criteria were used for model training, validation, and testing.

### 2.5. Model Architecture (ECG-PPG Fusion Network)

An ECG–PPG fusion neural network was designed with the primary goal of enabling real-time inference in wearable or edge-computing environments. The model consists of two parallel input pathways, one for ECG and one for PPG, which process synchronized 60 s signal segments (15,000 samples at 250 Hz) and extract modality-specific temporal features before multimodal fusion.

For each modality, temporal features were extracted using a temporal tokenizer, which is a hierarchical module designed to capture both local patterns and longer-range temporal contexts in physiological waveforms. The temporal tokenizer comprises two identical blocks. In each block, a standard 1D convolutional layer (32 filters, kernel size = 7) first learns the local patterns in the time domain. A kernel size of 7 corresponds to a time window of approximately 28 ms at 250 Hz, which is suitable for capturing key morphological features, such as the QRS complex in the ECG or the upstroke and peak of the PPG pulse wave. To improve computational efficiency while preserving representational power, convolution is implemented as a depth-wise separable convolution, in which temporal and channel-wise operations are factorized. This substantially reduces the number of trainable parameters compared to the standard convolution, which is critical for real-time inference on resource-constrained wearable devices.

Within each tokenizer block, a squeeze-and-excitation module is applied to model the interchannel dependencies and emphasize informative feature channels [48]. The SE block aggregates global information via global average pooling and learns channel-wise attention weights through two fully connected layers with a reduction ratio of eight, followed by sigmoid activation. The learned weights (ranging from 0 to 1) were multiplied by the original feature maps to selectively amplify physiologically relevant channels in the cardiovascular signals. The temporal resolution is then progressively reduced using average pooling (pool size = 5), which increases the receptive field and further improves computational efficiency. After passing through two blocks, the temporal resolution is progressively reduced: 15,000 input samples → 3000 tokens (after the first block) → 600 tokens (after the second block). Multiple tokens span each cardiac cycle, enabling the model to learn beat-level dynamics and preserve morphological and phase relationships for downstream fusion and regression. Consequently, multiple tokens cover a single cardiac cycle (≈800–1000 ms), enabling the model to explicitly encode beat-to-beat dynamics.

To learn the physiological interactions between ECG and PPG signals, a lightweight cross-attention mechanism was introduced on top of the tokenized representations. In this module, the ECG token sequence was projected onto the query (Q) vectors, whereas the PPG token sequence was projected onto the key (K) and value (V) vectors through independent linear layers (dimension = 32). The scaled dot-product attention is then computed as

(4) $$\mathrm{Attention}(Q,K,V)=\mathrm{softmax}(\frac{QK^{T}}{\sqrt{d_{k}}})V$$

where $d_{k}$ is the key dimension. For each ECG time token, this operation yields a weighted combination of all PPG tokens based on their similarity in the learned feature space. Physiologically, this configuration reflects the causal relationship between the ECG and PPG, which encode the timing of myocardial electrical activation, whereas the PPG reflects delayed peripheral flow changes. Cross-attention, therefore, allows the model to automatically learn which PPG features (e.g., delayed peaks and amplitude variations) should be referenced at each ECG time point to accurately infer the CO, effectively capturing phenomena such as pulse transit time and electromechanical coupling.

The outputs of the ECG tokenizer, PPG tokenizer, and cross-attention module were converted into fixed-length feature vectors (32 dimensions) via global average pooling along the temporal axis. Global average pooling is less sensitive to outliers than max pooling and aggregates information from the entire segment, making it suitable for summarizing the average characteristics of long physiological recordings. The three pooled vectors were then concatenated into a 96-dimensional fused feature vector that jointly encoded electrical activity (ECG), peripheral blood flow (PPG), and their learned interactions. This fused representation was passed through two fully connected layers (64 and 32 units) with rectified linear unit activation and dropout (rate = 0.3) to introduce nonlinearity and reduce overfitting. The final regression head consisted of a single linear neuron without activation, producing a continuous output corresponding to the predicted target (CO or CI, depending on the training configuration). The total number of trainable parameters is 33,745, ensuring the network remains sufficiently lightweight for real-time deployment on wearable or edge-computing platforms. The complete layer-by-layer architecture specification is provided in Supplementary Table S4 and Supplementary Figure S2.

### 2.6. Loss Function and Training Strategy

The loss function for model training was designed to simultaneously optimize prediction accuracy and concordance. The complete loss function is defined as:

(5) $$L=L_{\mathrm{Huber}}+\lambda \cdot (1-\mathrm{CCC})$$

where $L_{\mathrm{Huber}}\;$is the Huber loss with threshold δ = 1.0, CCC is the concordance correlation coefficient, and λ = 0.1 is a weighting factor that balances point prediction accuracy with concordance optimization.

Huber loss is a loss function that combines the advantages of the mean squared error (MSE) and mean absolute error (MAE), providing robustness to outliers. A threshold of δ=1.0 was used, applying squared loss when the error is less than δ and linear loss when greater, to limit the influence of extreme values:

(6) $$L_{\mathrm{Huber}}(\delta )=\left\{\begin{array}{c} \frac{1}{2}{\left(y-\hat{y}\right)}^{2}\;\mathrm{if}\;|y\;-\;\hat{y}|\;\leq \;\delta \;\\ \delta |y\;-\;\hat{y}|\;-\;\frac{1}{2}\delta^{2}\;\mathrm{if}\;|y\;-\;\hat{y}|\;\geq \;\delta \; \end{array}\right.$$

In physiological signal-based predictions, outliers can occur because of measurement errors, temporary signal quality degradation, or extreme physiological states. The Huber loss provides fast convergence, such as MSE for small errors, while increasing linearly, such as MAE for large errors, preventing outliers from excessively dominating the gradient updates.

The CCC-based term directly optimizes the concordance between predicted and actual values. The CCC considers not only simple linear correlation but also systematic bias and scale difference, making it suitable for evaluating the concordance of medical measurements.

(7) $$\mathrm{CCC}=\frac{2\sigma_{xy}}{\sigma_{x}^{2}+\sigma_{y}^{2}+(\mu_{x}-\mu_{y}{)}^{2}}$$

By including (1−CCC) in the loss function, the model is guided to achieve high concordance with actual values beyond simply reducing prediction error, and a weight of λ=0.1 was used to balance with Huber loss.

For numerical stability, $\epsilon =10^{-6}\;$was added to the denominator in CCC calculation, and the final CCC value was clipped to the range [−1, 1]. Clipping prevents the CCC from exceeding its theoretical range owing to numerical instability, particularly preventing numerical overflow or gradient explosion, which can occur when the variance is very small or the mean difference is large in batch-wise training. This composite loss function design aims to secure clinically meaningful concordance and learn a model that is robust to outliers, beyond simply reducing the prediction error.

Model training was performed using the Adam optimizer, with an initial learning rate set to $1\times 10^{-4}$. The Adam optimizer applies adaptive learning rates to each parameter and combines the advantages of momentum and adaptive learning rates to provide fast and stable convergence in deep neural networks. The batch size was set to 32 to balance memory efficiency and training stability, and training was conducted for a maximum of 1000 epochs.

Validation set-based monitoring was performed to prevent overfitting and select the optimal model. The early stopping technique was applied to terminate the training early if the validation loss did not improve for 20 epochs, and the weights at the point with the lowest validation loss were used as the final model. This effectively prevents overfitting situations in which the training loss continues to decrease, but the validation loss increases.

The learning process is optimized using learning-rate scheduling. The ReduceLROnPlateau (Reduce Learning Rate on Plateau) strategy was used, which reduces the learning rate by 0.5 times if the validation loss does not improve for 4 epochs, with the minimum learning rate limited to $1\times 10^{-6}$. This strategy of progressively reducing the learning rate enables fast convergence in the early stages of training and allows finding a better local minimum through fine-tuning in later stages.

Direct CO and CI prediction models were independently trained using the same model structure and training strategy. The two models had identical architectures, hyperparameters, and loss functions, with only the output targets set differently as CO and CI. The indirect CO prediction was calculated by multiplying the predicted value of the trained CI model by the patient-specific BSA (${\mathrm{CO}}_{\mathrm{indirect}}={\mathrm{CI}}_{\mathrm{pred}}\times \mathrm{BSA}$).

### 2.7. Statistical Analysis

Baseline characteristics were summarized descriptively as follows: continuous variables as medians with interquartile ranges and categorical variables as counts and percentages. The performance was assessed using the Pearson correlation coefficient (PCC), CCC, R^2^, MAE, root MSE (RMSE), and percentage error (PE). Confidence intervals (CoIs) are reported for all metrics. PCC CoIs used Fisher’s z-transformation. CoIs for CCC, R^2^, MAE, RMSE, and PE were computed using segment-level bootstrap resampling (2000 iterations with replacement from the test set). We acknowledge that segment-level bootstraps do not fully account for within-patient clustering due to overlapping segments and may underestimate uncertainty compared to patient-level hierarchical bootstraps. However, patient-level bootstrap was not feasible given the small test set size (n = 6 patients), which would result in high resampling variance. Clinical acceptability was examined using PE, defined as the ratio of the standard deviation of prediction errors to the mean reference value; a PE < 30% was considered acceptable. Agreement was evaluated with Bland–Altman analysis, defining bias as the mean difference (d = ŷ − y) and the 95% limits of agreement (LoA) as bias ± 1.96 × SD(d), with CoIs derived from standard formulas. We primarily report Bland–Altman results in absolute units (L/min). For interpretability, percent bias and percent LoA were additionally reported by normalizing to the mean reference CO across the evaluated set (ȳ_ref = mean(y)): percent bias = 100 × bias/ȳ_ref and percent LoA = 100 × (LoA_lower, LoA_upper)/ȳ_ref. To compare direct CO prediction with indirect CO (CI × BSA), we tested paired differences: PCC via a paired permutation test (5000 permutations); CCC and R^2^ via bootstrap (3000 resamples); and MAE, RMSE, and bias using nonparametric tests after Shapiro–Wilk normality checks. Statistical significance was set at *p* < 0.05 indicated significance. All preprocessing, statistical analysis, and modeling used Python 3.9 with NumPy, Pandas, SciPy, Scikit-learn, TensorFlow/Keras, and NeuroKit2.

## 3. Results

### 3.1. Baseline Characteristics

Twenty-seven adult patients who underwent cardiac surgery were analyzed. Table 1 summarized the baseline characteristics. Median age was 70.0 years (60.0–73.5); 17 (63.0%) were male. Median height, weight, body mass index (BMI), and BSA were 167.0 cm (155.0–168.5), 63.0 kg (58.0–69.0), 24.1 kg/m^2^ (22.2–25.7), and 1.7 m^2^ (1.6–1.8). Preoperative echocardiography showed preserved systolic function (median Left Ventricular Ejection Fraction (LVEF) 67% [58.5–70.0]). The primary diagnoses included aortic valve regurgitation (18.5%), ascending aortic aneurysm (18.5%), aortic valve stenosis (14.8%), abdominal aortic aneurysm (14.8%), aortic arch aneurysm, dissection, abdominal aortic occlusive disease, and peripheral artery occlusive disease. The major procedures were aortic valve replacement (33.3%), graft replacement of the ascending aorta (25.9%) with additional abdominal/descending aortic grafts, mitral valve replacement, subaortic myectomy, and aortofemoral bypass. Intraoperative ventilation used a peak inspiratory pressure of 16 cmH_2_O (13–16), positive end-expiratory pressure of 5 cmH_2_O (5–7), and a tidal volume of 460 mL (409–530). Baseline hemodynamics were as follows: systolic blood pressure, 114 mmHg (100–130); diastolic blood pressure, 56 mmHg (47–62); and heart rate, 61 bpm (57–68). Reference CO and CI were 3.5 L/min (3.0–4.1) and 2.1 L/min/m^2^ (1.8–2.4) and the min-max ranges were 1.8–6.3 L/min and 1.3–4.2 L/min/m^2^, respectively

### 3.2. Performance of CI Prediction Model

**Figure 1:**
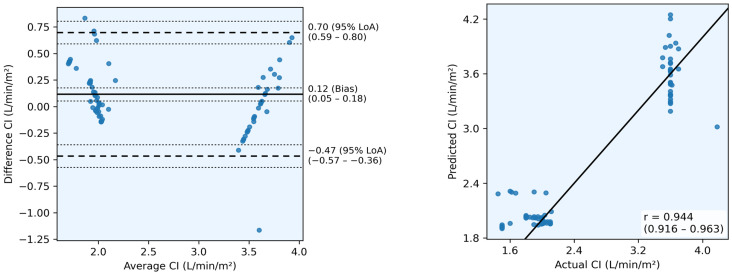
Cardiac index (CI) prediction performance using scatter plots (**left**) and Bland-Altman plots (**right**). In the scatter plot, the solid diagonal line represents perfect agreement (y = x). In the Bland-Altman plot, the solid horizontal line indicates mean bias, dashed lines represent 95% limits of agreement (LoA), and thin dashed lines show 95% confidence intervals.

Table 2 and Figure 1 summarize the performance of the CI prediction model, which serves as an intermediate step in indirect CO estimation. The model achieved excellent agreement with invasive references (PCC = 0.944, 95% CoI 0.916–0.963; CCC = 0.929, 95% CoI 0.898–0.951; R^2^ = 0.871, 95% CoI 0.824–0.910). The MAE was 0.241 L/min/m^2^ (95% CoI, 0.201–0.285), and the RMSE was 0.317 L/min/m^2^ (95% CoI 0.261–0.377). The mean PE was 23.5%, which met the clinical acceptability threshold of PE < 30%. Bland–Altman analysis showed a minimal bias (0.12 L/min/m^2^) and narrow 95% LoA (−0.47 to 0.70 L/min/m^2^). These results indicate that the model accurately predicted CI values and explained most of the variance in invasive measurements, supporting its use in indirect, noninvasive CO estimation.

### 3.3. Model Performance: Direct vs. Indirect CO Prediction

**Figure 2:**
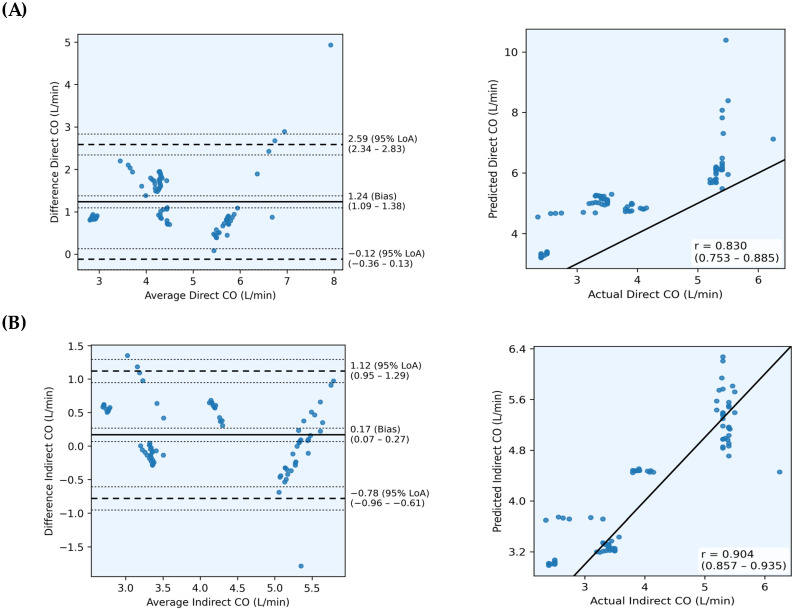
Comparison of model-predicted and reference cardiac output values using scatter plots and Bland-Altman plots. (**A**) Direct CO prediction; (**B**) Indirect CO prediction (via CI × BSA). In scatter plots, the solid diagonal line represents perfect agreement (y = x). In Bland-Altman plots, the solid horizontal line indicates mean bias, dashed lines represent 95% limits of agreement (LoA), and thin dashed lines show 95% confidence intervals. The indirect CO prediction model exhibited higher correlation, lower bias, and narrower limits of agreement compared with the direct model.

Table 3 and Figure 2 compare the performances of the direct and indirect CO prediction methods. Overall, the indirect approach, reconstructing CO as a predicted CI × BSA, improved the direct regression. The indirect model achieved strong agreement with the reference CO (PCC = 0.904, CCC = 0.886, R^2^ = 0.794) and low errors (MAE = 0.403 L/min, RMSE = 0.511 L/min, PE = 23.75%), thereby satisfying the clinical acceptability threshold (PE < 30%). Bland–Altman analysis showed a small bias (0.17 L/min) and narrow 95% LoA (−0.78 to 1.12), indicating clinically reliable performance. In contrast, the direct model, trained to regress CO directly from ECG–PPG inputs, exhibited reduced accuracy and stability (PCC = 0.831, CCC = 0.532, R^2^ = −0.575, MAE = 1.236 L/min, RMSE = 1.413 L/min, PE = 33.74%), with a high bias (1.24 L/min) and wide agreement limits. The negative R^2^ for the direct CO model indicates performance worse than a mean-value baseline predictor, suggesting poor fit under this evaluation setting. Statistically significant improvements in the indirect CO prediction were observed for MAE, RMSE, and bias, whereas PCC, CCC, R^2^, and PE values were not significantly different between models.

These findings confirm that CI-based normalization improved noninvasive CO estimation, yielding better correlation, concordance, and error profiles. Incorporating body size normalization improves generalizability across subjects and supports continuous wearable hemodynamic monitoring without invasive catheters.

## 4. Discussion

### 4.1. Principle Findings

In this prospective study of cardiac surgery patients monitored using pulmonary artery catheters, we developed and validated a lightweight deep learning model for noninvasive CO estimation using wearable ECG and PPG signals. Three key findings emerged. First, the multimodal ECG–PPG fusion network accurately predicted the CI with strong correlation and concordance, achieving a PE below the predefined 30% clinical threshold [26]. Second, these findings suggest that CI-guided indirect CO reconstruction primarily improves absolute error and agreement (MAE/RMSE/bias), whereas gains in correlation-based metrics were not statistically confirmed in this cohort. Given the modest sample size, external validation in larger and more diverse settings will be required to confirm robustness across correlation and PE endpoints. Third, the compact, parameter-efficient design (<50 k parameters) of the model supports its potential integration into wearable or edge-computing platforms. Overall, the CI-based normalization proved to be an effective strategy for improving generalization and achieving clinically acceptable agreement with invasive CO measurements, thereby enabling continuous catheter-free hemodynamic monitoring.

### 4.2. Comparison with Existing Cardiac Output Monitoring Methods

Pulmonary artery catheterization remains the reference method for CO measurement. However, it is invasive, labor-intensive, and limited to critical care settings. Minimally invasive pulse contour systems require arterial lines and calibration, whereas echocardiography is intermittent and operator dependent. In contrast, the proposed wearable ECG–PPG approach enables continuous, noninvasive monitoring using compact, widely available devices. The indirect CO model achieved clinically acceptable accuracy (PE < 30%) and narrow Bland–Altman limits, comparable to adjunctive hemodynamic tools. Unlike previous noninvasive techniques, such as impedance cardiography, tonometry, or single-signal PPG, our multimodal fusion network exploits the physiological complementarity of ECG and PPG through cross-attention and CI normalization, enhancing stability, reducing interindividual variability, and improving agreement with invasive CO measurements across diverse clinical conditions.

To contextualize our agreement results, prior method-comparison studies of noninvasive CO monitoring often interpret Bland–Altman PE using the commonly cited ~30% benchmark, although this threshold depends on the precision of the reference technique and should be interpreted cautiously [49]. In our study, the indirect CO reconstruction (CI × BSA) showed a bias of 0.17 L/min with PE 23.75% and 95% LoA of −0.78 to 1.12 L/min (Table 3). These values are comparable to favorable reports for finger-cuff volume clamp pulse contour monitoring in cardiac surgery patients (e.g., ClearSight showing bias ~0.32–0.36 L/min with PE ~25–26% in one PAC-thermodilution comparison) [50], although performance can deteriorate in certain pathophysiologic conditions (e.g., severe aortic stenosis with PE > 40%) [51]. In contrast, ECG/PPG PWTT-based esCCO has frequently demonstrated wider LoA and higher PE in perioperative comparisons (e.g., intraoperative PE ~64% in an off-pump coronary artery bypass cohort; PE ~46% in another major-surgery comparison) [36,37] and thoracic bioimpedance/bioreactance approaches have also reported high PE in challenging surgical settings (e.g., NICOM CI PE > 60% in liver transplantation) [52]. Similarly, minimally invasive arterial pressure waveform/pulse contour system (e.g., FloTrac) may show substantial PE in low-SVR states and certain surgeries (often >50–60%) [53,54,55]. Collectively, these benchmarks suggest that our wearable ECG-PPG fusion approach achieves competitive agreement under controlled periperative conditions, while broader validation across diverse hemodynamic and artifact-rich environments remains necessary.

### 4.3. Advantages of Cardiac Index-Based Indirect Prediction

One of the key findings of this study was that indirect CO estimation through CI prediction improved direct CO regression. CI normalization by BSA reduces interindividual variability due to body size differences, allowing the model to focus on waveform-derived physiological patterns rather than anthropometric variability. From a machine-learning perspective, CI normalization constrains the prediction target to a narrower, more homogeneous range, mitigating the influence of extreme values that can increase loss variance and hinder generalization. Consequently, the CI model showed superior concordance and fewer errors than the direct CO regression. When the CO was reconstructed from the predicted CI and BSA, the network benefited from both physiological normalization and personalized scaling, achieving a stronger agreement with the invasive CO. These results highlight the robustness and physiological soundness of a two-step approach—predicting CI from biosignals and deriving CO via BSA multiplication—for noninvasive CO estimation.

### 4.4. Clinical Implications and Potential Applications

The proposed framework may have potential clinical utility as a noninvasive adjunct for continuous hemodynamic monitoring across selected care settings. In perioperative care, a wearable ECG–PPG system could provide continuous CO/CI estimates before and after surgery, particularly when invasive monitoring is unavailable, impractical, or has been discontinued. In intensive care and step-down units, such estimates may complement conventional monitoring by providing real-time trends that could help identify early physiologic changes and support decisions such as catheter weaning, when interpreted alongside other vital signs and clinical assessment. Beyond the hospital, patch-based ECG and finger PPG devices used in chronic heart failure or pulmonary hypertension might eventually incorporate CO/CI estimation for home monitoring; however, this application remains untested in the present study and would require dedicated validation in ambulatory, motion-rich conditions. Using only ECG and PPG may also facilitate integration into existing wearable ecosystems. Importantly, this approach is not intended to replace invasive CO measurements, but rather to extend hemodynamic insight to less critical settings by providing clinically meaningful trend information.

Clinical interpretation of error metrics is important for practical use. In our study, the CI MAE (~0.24 L/min/m^2^) represents an absolute error of roughly ~6–10% relative to typical adult CI ranges (2.5–4.0 L/min/m^2^), and the CO MAE (~0.40 L/min) corresponds to ~5–10% relative to typical adult CO ranges (4–8 L/min) [56]. While these errors appear modest in magnitude, they may still be clinically relevant when value lies near commonly used low-output thresholds (e.g., CI ≈ 2.2 L/min/m^2^), where small absolute differences can affect classification [57,58]. Accordingly, the present model is best viewed as an adjunct for continuous noninvasive monitoring and trend, rather than a stand-alone tool for immediate therapeutic titration based on single-point estimates. Prospective validation in broader, more heterogenous conditions is required before considering direct decision-making use.

### 4.5. Limitations and Future Directions

This study has several limitations. First, the cohort was small and derived from a single center, which limits the generalizability of our findings. In addition, the absence of an external validation dataset is a significant constraint, particularly for medical applications where model performance may vary across institutions, patient populations, and clinical workflows. Multicenter external validation in diverse clinical contexts is essential before this approach can be considered for broader use. Second, the dataset was restricted to patients undergoing mainly aortic cardiac surgery under general anesthesia with controlled ventilation and relatively stable perioperative conditions. Accordingly, our cohort may not represent broader cardiac-surgery populations or patients with reduced LVEF, who may exhibit different hemodynamic states and ECG/PPG waveform characteristics that could affect model performance. In addition, all data were collected in a controlled perioperative environment with minimal motion; thus, performance in awake or ambulatory settings—where posture changes (e.g., supine-to-standing) and motion artifacts are common—or in more heterogeneous and unstable physiologic states (e.g., arrhythmias, low-perfusion states, shock, or critical illness) remains unknown. Moreover, recordings covered short intraoperative periods with mostly stable physiology; therefore, performance during dynamic transitions, interventions, or rapid hemodynamic perturbations was not evaluated. Future work will include validation in more diverse populations (including reduced LVEF) and prospective testing in ambulatory, artifact-rich conditions. Third, although data partitioning was performed at the patient level to prevent information leakage across training, validation, and test sets, we extracted 60 s windows with 50% overlap within each partition. This overlap introduces within-subject temporal correlation and statistical dependence among segments. While overlap increases data efficiency in a limited cohort, segment-level resampling can underestimate uncertainty compared with patient-level (cluster) resampling. This limitation primarily affects uncertainty quantification rather than model validity, because the held-out test set remained independent at the patient level. Patient-level cross-validation was not adopted in this study due to the small number of subjects, which would yield very small test folds and unstable performance estimates. Future studies with larger multicenter cohorts should employ patient-level resampling and subject-level cross-validation (e.g., leave-one-subject-out) to more robustly characterize performance and uncertainty. Fourth, the model used only ECG and PPG inputs. Incorporating additional signals (e.g., arterial blood pressure waveforms, respiration, or temperature) and/or relevant clinical variables may improve accuracy and robustness. Comparative evaluations against alternative architectures and simpler baselines in larger datasets are also warranted. Fifth, we did not evaluate downstream clinical outcomes; therefore, interventional studies are needed to determine whether model-guided monitoring improves patient management or outcomes. Finally, computational efficiency and real-time performance on wearable hardware were not fully assessed and should be examined in future work. Despite these limitations, our results support the feasibility of CI-based indirect CO estimation using wearable ECG–PPG fusion under controlled perioperative conditions, motivating further validation in larger and more diverse settings.

## 5. Conclusions

In this prospective study of cardiac surgery patients monitored with PAC, we developed a lightweight deep learning model that noninvasively estimates CO from wearable ECG and PPG signals. By training the network to predict the CI and reconstruct the CO as the product of the predicted CI and BSA, the proposed indirect approach achieved substantially better agreement with invasive reference measurements than direct CO regression. The proposed noninvasive ECG–PPG fusion approach met the predefined agreement benchmark (PE < 30%) for indirect CO estimation. These results support feasibility for continuous, catheter-free monitoring and trend assessment; however, the approach is not intended to replace PAC-based measurements for definitive hemodynamic assessment. Multicenter external validation and prospective evaluation in ambulatory, motion-rich, arrhythmic, and low-perfusion conditions are required before broader clinical deployment.

These findings demonstrate that multimodal ECG–PPG fusion combined with CI–based normalization can provide clinically acceptable CO estimates using only two widely available wearable signals. This study lays the foundation for future studies to validate and extend this framework across broader patient populations and care settings, ultimately enabling continuous catheter-free hemodynamic monitoring.

## Figures and Tables

**Table 1:** Baseline Characteristics of Participants.

	Total (n = 27)
Age (y, median, IQR)	70.0 (60.0, 73.5)
Male (n (%))	17 (63.0)
Height (cm, median, IQR)	167.0 (155.0, 168.5)
Weight (kg, median, IQR)	63.0 (58.0, 69.0)
BMI (kg/m^2^, median, IQR)	24.1 (22.2, 25.7)
BSA (m^2^, median, IQR)	1.7 (1.6, 1.8)
Preop-echo (Ejection Fraction (%), median, IQR)	67 (58.5, 70)
Diagnosis for admission (n (%))	
Aortic valve regurgitation	5 (18.5)
Aortic valve stenosis	4 (14.8)
Mitral valve regurgitation	1 (3.7)
Mitral valve stenosis	1 (3.7)
Ascending aorta aneurysm	5 (18.5)
Aortic arch aneurysm	2 (7.4)
Abdominal aorta aneurysm	4 (14.8)
Aortic dissection	2 (7.4)
Abdominal aorta occlusion disease	1 (3.7)
Peripheral artery occlusive disease	2 (7.4)
Ventilator setting during operation (median, IQR)	
Mode	PRVC
Peak inspiratory pressure (cmH_2_O)	16 (13, 16)
PEEP (cmH_2_O)	5 (5, 7)
Tidal volume (mL)	460 (409, 530)
Operation name (n (%))	
Aortic valve replacement	9 (33.3)
Mitral valve replacement	1 (3.7)
Graft replacement of ascending aorta	7 (25.9)
Graft replacement of descending aorta	2 (7.4)
Graft replacement of abdominal aorta	5 (18.5)
Subaortic fibromectomy	1 (3.7)
Aorto-femoral bypass	2 (7.4)
Hemodynamic parameters	
Systolic blood pressure (mmHg, median, IQR)	114.0 (100.0, 130.0)
Diastolic blood pressure (mmHg, median, IQR)	56.0 (47.0, 62.0)
Heart rate (/min, median, IQR)	61.0 (57.0, 68.0)
Actual CO (L/min, median, IQR) by PAC	3.5 (3.0, 4.1)
Actual CO (L/min, min-max) by PAC	1.8–6.3
Actual CI (L/min/m^2^, median, IQR) by PAC	2.1 (1.8, 2.4)
Actual CI (L/min/m^2^, min-max) by PAC	1.3–4.2

BMI, body mass index; BSA, body surface area; PRVC, pressure-regulated volume control; PEEP, positive end-expiratory pressure; CO, cardiac output; CI, cardiac index.

**Table 2:** Cardiac Index prediction.

Metric	CI Prediction
Pearson correlation coefficient (PCC)	0.944 [95% CoI 0.916, 0.963]
Concordance correlation coefficient (CCC)	0.929 [95% CoI 0.898, 0.951]
Coefficient of determination (R^2^)	0.871 [95% CoI 0.824, 0.910]
MAE (L/min/m^2^)	0.241 [95% CoI 0.201, 0.285]
RMSE (L/min/m^2^)	0.317 [95% CoI 0.261, 0.377]
Mean percentage error (%)	23.52 [95% CoI 18.80, 28.79]
Clinical acceptability (PE < 30%)	Satisfied
Bias (L/min/m^2^)	0.12 [95% CoI 0.05, 0.18]
95% limits of agreement (L/min/m^2^)	−0.47 (95% CoI −0.57, −0.36) to 0.70 (95% CoI 0.59, 0.80)

MAE, mean absolute error; RMSE, root mean squared error; PE, percentage error; CoI, confidence interval.

**Table 3:** Comparison of Model Performance for Cardiac Output (CO) and Cardiac Index (CI) Prediction.

Metric	Direct CO Prediction	Indirect CO Prediction (via CI × BSA)	*p*-Value (Direct vs. Indirect)
Pearson correlation coefficient (PCC)	0.830 [95% CoI 0.753, 0.885]	0.904 [95% CoI 0.857, 0.935]	0.2503
Concordance correlation coefficient (CCC)	0.534 [95% CoI 0.457, 0.611]	0.886 [95% CoI 0.841, 0.917]	0.5142
Coefficient of determination (R^2^)	−0.575 [95% CoI −1.202, −0.123]	0.794 [95% CoI 0.724, 0.848]	0.5232
MAE (L/min)	1.236 [95% CoI 1.102, 1.380]	0.403 [95% CoI 0.341, 0.470]	<0.0001
RMSE (L/min)	1.413 [95% CoI 1.226, 1.631]	0.511 [95% CoI 0.430, 0.600]	<0.0001
Mean percentage error (%)	33.74 [95% CoI 24.77, 43.60]	23.75 [95% CoI 19.49, 28.59]	0.4605
Clinical acceptability (PE < 30%)	Not satisfied	Satisfied	-
Bias (L/min)	1.24 [95% CoI 1.09, 1.38]	0.17 [95% CoI 0.07, 0.27]	<0.0001
95% limits of agreement (L/min)	−0.12 (95% CoI −0.36, 0.13) to 2.59 (95% CoI 2.34, 2.84)	−0.78 (95% CoI −0.96, −0.61) to 1.12 (95% CoI 0.95, 1.30)	0.4605

MAE, mean absolute error; RMSE, root mean squared error; PE, percentage error; CoI, confidence interval.

## Data Availability

Data from this study are available upon request after publication by contacting the corresponding author (chungks@yuhs.ac). The data will be shared with those who submit a well-structured proposal based on sound methodology. Patient data were anonymized and temporal data were adjusted within a randomly selected period in adherence to the HIPAA guidelines.

## Supplementary Materials

The following supporting information can be downloaded at: https://www.mdpi.com/article/10.3390/s26020735/s1, Supplementary Table S1: Specifications of the HiCardi+ devices, Supplementary Table S2: Specifications of the WristOx2 3150 devices, Supplementary Table S3: Dataset composition and partition overview, Supplementary Table S4: Detailed Model Architecture Specification, Supplementary Figure S1: Distribution of reference Cardiac output values obtained from the Pulmonary Artery Catheter across all study participants, Supplementary Figure S2: Model architecture using ECG-PPG fusion.

## Abbreviations

The following abbreviations are used in this manuscript:

ECG	ElectroCardioGraphy
PPG	PhotoPlethysmoGraphy
CO	Cardiac Output
CI	Cardiac Index
CoI	Confidence of Interval
SpO_2_	Oxygen saturation
BSA	Body Surface Area
BMI	Body Mass Index
MSE	Mean Squared Error
RMSE	Root Mean Squared Error
CCC	Concordance Correlation Coefficient
LoA	Limits of Agreement
LVEF	Left Ventricular Ejection Fraction
PWTT	Pulse-Wave-Transit-Time
esCCO	Estimated Continuous Cardiac Output
PE	Percentage Error
