# Supplementary material for: Wearable ECG-PPG Deep Learning Model for Cardiac Index-Based Noninvasive Cardiac Output Estimation in Cardiac Surgery Patients
Source: Sensors (Basel). 2026 Jan 22;26(2):735. doi: 10.3390/s26020735 (PMC12845561; doi:10.3390/s26020735)

Supplementary Table S1. Specifications of the HiCardi+ devices

Detailed technical specifications of the HiCardi+ single-lead ECG chest-patch developed by MEZOO Co., Ltd. The table summarizes hardware parameters, sampling rate, resolution, wireless communication, power supply, data storage, and special features, including its real-time ECG streaming and arrhythmia-detection capabilities.

Parameter	Specification
Device name	HiCardi+
Manufacturer	MEZOO Co., Ltd. (Wonju, Republic of Korea)
Device type	Wireless, single-channel ECG chest-patch (smart-patch–based wearable ECG monitor)
Measured signals	1-lead ECG, respiration, skin temperature, activity/posture, step/movement
Sampling rate (ECG)	250Hz
Resolution	16 bits
Communication	Bluetooth Low Energy (BLE)
Power supply	Rechargeable battery (approximately 72 h operation)
Data storage	Cloud-connected via BLE gateway or mobile app
Weight (including battery) / Size	~18g / 59×35.5×8.5 mm
Special features	Real-time ECG streaming; automatic detection of 17 arrhythmia types; geometric-angle–based R-peak detection algorithm; robust against QRS morphology variation and baseline drift

Supplementary Table S2. Specifications of the WristOx2 3150 devices

Comprehensive specifications of the WristOx2 3150 pulse oximeter used for photoplethysmography (PPG) signal acquisition. Key features include dual-channel PPG, SpO_2 and pulse-rate measurement, sampling characteristics, power consumption, and PureSAT® motion-tolerant signal processing validated for continuous clinical monitoring.

Parameter	Specification
Device name	WristOx2 Model 3150
Manufacturer	Nonin Medical, Inc. (Plymouth, MN, USA)
Device type	Wrist-worn pulse oximeter with finger sensor (wearable photoplethysmography-based sensor)
Measured signals	Photoplethysmography (PPG; infrared and red channels), oxygen saturation (SpO_2), pulse rate
Sampling rate (PPG)	75 Hz
Resolution	16 bits
Communication	Bluetooth Low Energy (BLE) / USB
Power supply	Two AAA batteries; continuous use with BLE ≈ 44 h (alkaline, new); without BLE ≈ 53 h.
Data storage	Internal memory (up to 1,080 h) and PC data download via nVision® software
Weight / Size	~71 g / 46 × 69 × 31 mm
Special features	Clinically validated for continuous SpO_2 monitoring; motion-tolerant PureSAT® signal processing; suitable for hospital and ambulatory use

Supplementary Table S3. Dataset composition and partition overview

Summary of dataset division into training, validation, and test sets at the patient level. The table describes the number of subjects and signal segments per subset and the corresponding proportion used for model training, hyperparameter tuning, and final performance evaluation to avoid inter-patient information leakage.

Subset	Number of Patients (n)	Number of Segments (n)	Proportion (%)	Description
Training set	16	302	59.26	Used for model training
Validation set	5	108	18.52	Used for hyperparameter tuning and model selection
Test set	6	91	22.22	Used for final model evaluation
Total	27	501	100.00	

Supplementary Table S4. Detailed Model Architecture Specification

Layer Type	Output Shape	Parameters	Description
ECG Input	(None, 15000, 1)	0	Single-lead ECG waveform
PPG Input	(None, 15000, 1)	0	Finger PPG waveform
Conv1D (ECG/PPG)	(None, 15000, 32)	256 each	Initial feature extraction
SeparableConv1D (ECG/PPG)	(None, 15000, 32)	1,280 each	Depthwise separable convolution
SE Block 1 (ECG/PPG)	(None, 15000, 32)	292 each	Channel attention (reduction ratio=8)
AveragePooling1D	(None, 3000, 32)	0	Temporal downsampling (pool_size=5)
Conv1D (ECG/PPG)	(None, 3000, 32)	7,200 each	Second-stage feature extraction
SeparableConv1D (ECG/PPG)	(None, 3000, 32)	1,280 each	Depthwise separable convolution
SE Block 2 (ECG/PPG)	(None, 3000, 32)	292 each	Channel attention (reduction ratio=8)
AveragePooling1D	(None, 600, 32)	0	Temporal downsampling (pool_size=5)
Cross-Attention	(None, 600, 32)	4,224	ECG-PPG interaction learning
GlobalAveragePooling1D	(None, 32) × 3	0	Temporal aggregation
Concatenate	(None, 96)	0	Multi-pathway fusion
Dense	(None, 64)	6,208	Fully connected layer
Dropout	(None, 64)	0	Regularization (rate=0.3)
Dense	(None, 32)	2,080	Fully connected layer
Output Dense	(None, 1)	33	Regression output (CO or CI)
**Total**	**-**	**33,745**	**All parameters trainable**

Note: The temporal tokenizer consists of two identical blocks (Conv1D → SeparableConv1D → SE → Pooling), progressively reducing temporal resolution from 15,000 → 3,000 → 600 tokens while extracting hierarchical time-dependent features.

Supplementary Figure S1. Distribution of reference Cardiac output values obtained from the Pulmonary Artery Catheter across all study participants

Histogram illustrating the distribution of reference cardiac output (CO) and cardiac index (CI) measured via the pulmonary artery catheter, body surface area, and heart rate across all study participants. The figure depicts inter-subject variability and the range of hemodynamic states represented in the dataset, serving as a reference for model prediction accuracy.

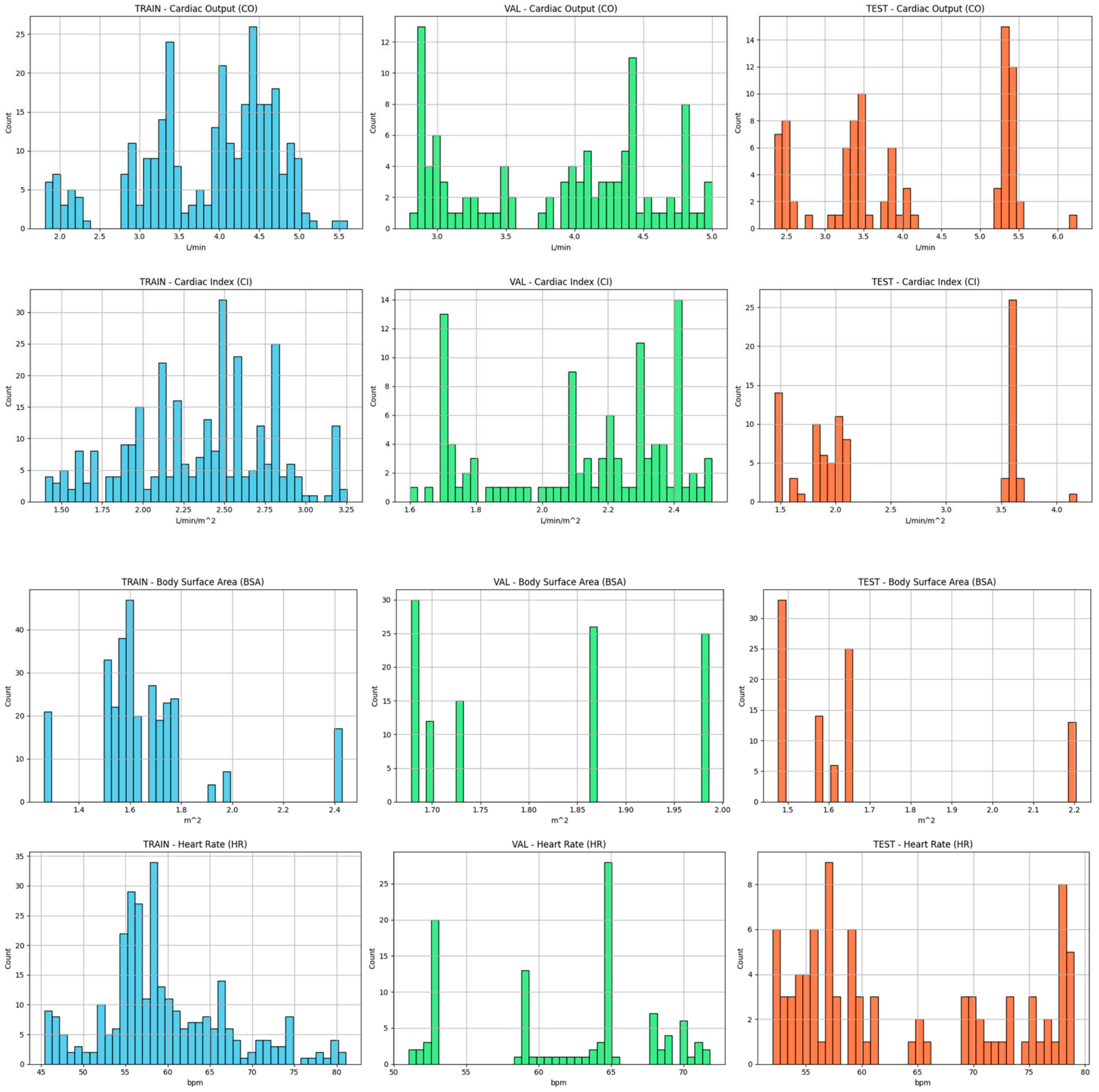

Supplementary Figure S2. Model architecture using ECG-PPG fusion

Schematic of the proposed lightweight fusion model for real-time cardiac output estimation. Parallel temporal tokenizers extract beat-level features from ECG and PPG signals, integrated through cross-attention to capture electromechanical coupling. The concatenated embeddings are passed through dense layers to yield CO or CI predictions with <50 k parameters for edge deployment.

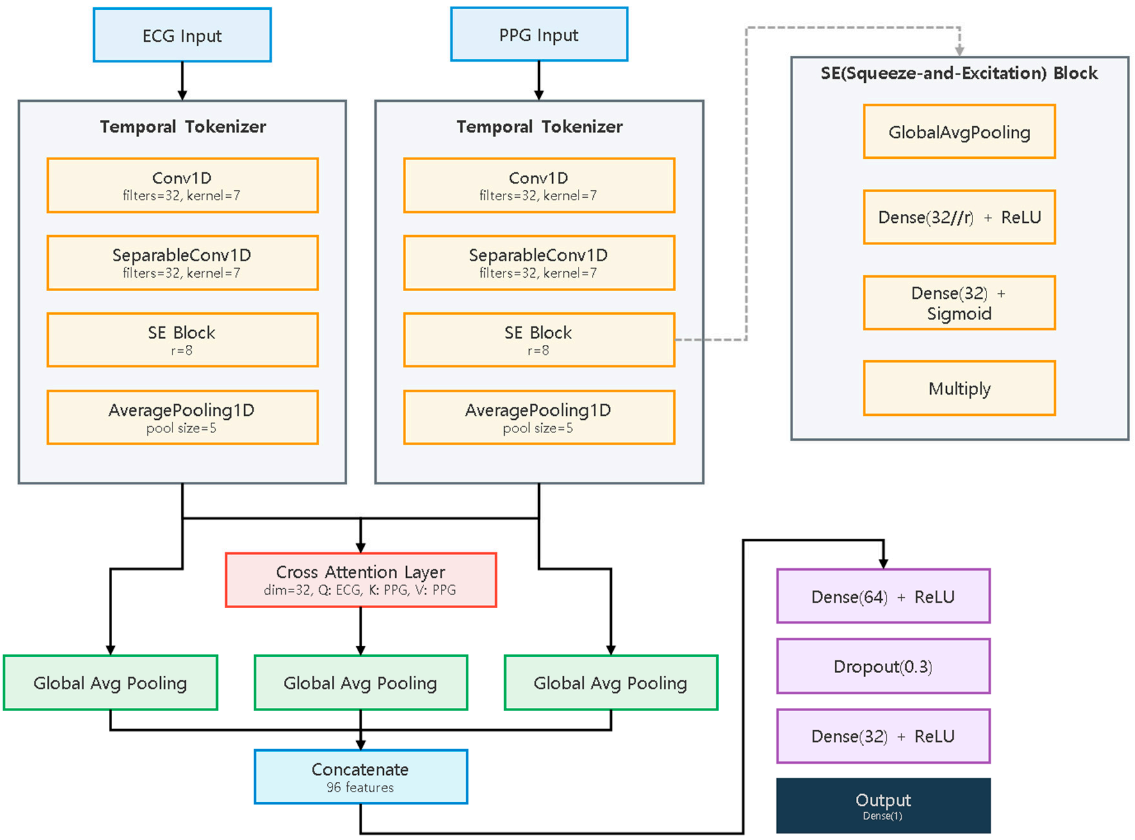

Supplement: Supplementary file 1 [file sensors-26-00735-s001.zip › sensors-4029166-supplementary.pdf]
